# Jigless knotless internal brace technique for acute Achilles tendon rupture: a case series study

**DOI:** 10.1186/s13018-019-1471-8

**Published:** 2019-12-05

**Authors:** Po-Yen Ko, Ming-Tung Huang, Chia-Lung Li, Wei-Ren Su, I-Ming Jou, Po-Ting Wu

**Affiliations:** 1 Department of Biomedical Engineering, National Cheng Kung University, Tainan, Taiwan; 2 Department of Orthopedics, National Cheng Kung University Hospital Dou-Liu Branch, College of Medicine, National Cheng Kung University, Yunlin, Taiwan; 3 Department of Orthopedics, National Cheng Kung University Hospital, College of Medicine, National Cheng Kung University, Tainan, Taiwan; 4 Department of Orthopedics, College of Medicine, National Cheng Kung University, 1 University Road, East District, Tainan City, 701 Taiwan; 5 Department of Orthopedics, E-Da Hospital, Kaohsiung, Taiwan; 6 School of Medicine, College of Medicine, I-Shou University, Kaohsiung, Taiwan; 7 Medical Device Innovation Center, National Cheng Kung University, Tainan, Taiwan

**Keywords:** Achilles tendon rupture, Minimally invasive, Knotless, Internal brace

## Abstract

**Purpose:**

To mitigate the risk of poor wound healing and of infection associated with the open repair of Achilles tendon midsubstance ruptures, minimally invasive techniques have been developed. We report our preliminary results after reviewing our “jigless knotless internal brace technique.”

**Methods:**

Patients were placed in prone position and a transverse 3-cm incision was made proximal to the palpable ruptured end. The proximal ruptured end was pulled out, gently debrided, and sutured using Krackow locking loops. Percutaneous sutures were crisscrossed through the distal tendon stump and looped around the Krackow sutures over the proximal stump. The ipsilateral Krackow sutures and the contralateral crisscrossed sutures were subcutaneously passed through two mini-incisions over the posterior calcaneus tuberosity and seated at the tuberosity with two 4.5-mm knotless suture anchors. All patients underwent the same post-operative rehabilitation protocol and regular follow-ups for at least 1 year.

**Results:**

We recruited 10 patients (mean age, 37.3 years) who scored 100 points on the American Orthopaedic Foot and Ankle Society (AOFAS) scale, and who returned to their preoperative exercise levels 1-year post-operatively with no complications.

**Conclusion:**

Our method is simple, effective, and requires no special tools. It might be a reliable option for Achilles tendon repair.

**Level of evidence:**

III

## Introduction

Although the Achilles tendon is the human body’s strongest tendon, it is also the tendon most frequently ruptured [1]. Over the past quarter-century, the rupture incidence rate has significantly increased [2–4]. A Danish national study [3] reported more than 33,000 ruptures (males 47/100,000; females 17/100,000).

Achilles tendon management options have been reported: non-operative management with a short leg-cast, a brace in an equinus position, and surgical repair [4–6]. There was no consensus on the best option [7–10]. Some studies have reported lower rerupture rates in operative groups [11, 12], but others have claimed nearly identical rerupture rates in both operative and non-operative groups [13]. For active young athletes who must quickly return to competition, surgery is indicated to avoid muscle atrophy after non-operative management [14]. There are several operative Achilles repair methods: percutaneous and minimally invasive surgery (MIS), and open repair with or without augmentation. One high-quality meta-analysis [15] reported no significant differences in deep infection, rerupture, tissue adhesion, or nerve injury rates between minimally invasive surgery (MIS) and open surgery; MIS, however, has better subjective outcomes and a significantly lower superficial infection rate [15]. Despite these benefits, injuring the sural nerve during MIS is still a risk [16–18].

A recent cadaver study reported that the sural nerve would crosses the lateral border of the Achilles tendon 8- to 10-cm proximal to the superior border of the calcaneal tuberosity in most cases [19]. Therefore, a surgeon can avoid injuring the sural nerve by doing all percutaneous procedures within 8 cm proximal to the calcaneal tuberosity. Hence, we have developed a novel Achilles tendon surgical method called the “jigless knotless internal brace technique” to repair the Achilles tendon. We report our preliminary results using this technique.

## Materials and methods

### Patients

Written informed consent was obtained from all patients. All procedures were approved by the National Cheng Kung University Hospital’s (NCKUH) Institutional Review Board. Consecutive patients treated for acute Achilles tendon injury at NCKUH between January 2015 and July 2017were evaluated. Our inclusion criteria were a positive Simmonds test (aka Thompson test or Simmonds-Thompson test) and a palpable defect in the Achilles tendon corresponding to a midsubstance rupture. Surgery occurred within 2 weeks post-injury. The exclusion criteria were a cutting or penetrating injury, an injury more than 2 weeks old, a neurological or a psychiatric disorder, pregnancy, being less than 18 years old, an incomplete medical record, an inadequate follow-up, autoimmune or connective tissue diseases (e.g., rheumatoid arthritis), radiotherapy or chemotherapy, morbid obesity, and previous Achilles tendon surgery. Finally, 10 patients (men, 9; women, 1; minimum follow-up, 1 year; mean age, 37.3 years; age range, 20–53 years; mean body mass index [BMI], 24.5; BMI range, 22.1–29.7) were enrolled. All enrolled patients had undergone the same post-operative rehabilitation protocol, and all returned to our hospital for clinical follow-ups 2, 4, 6, and 8 weeks, and 3, 4, 6, and 12 months post-surgery.

### Surgical technique

**Fig. 1:**
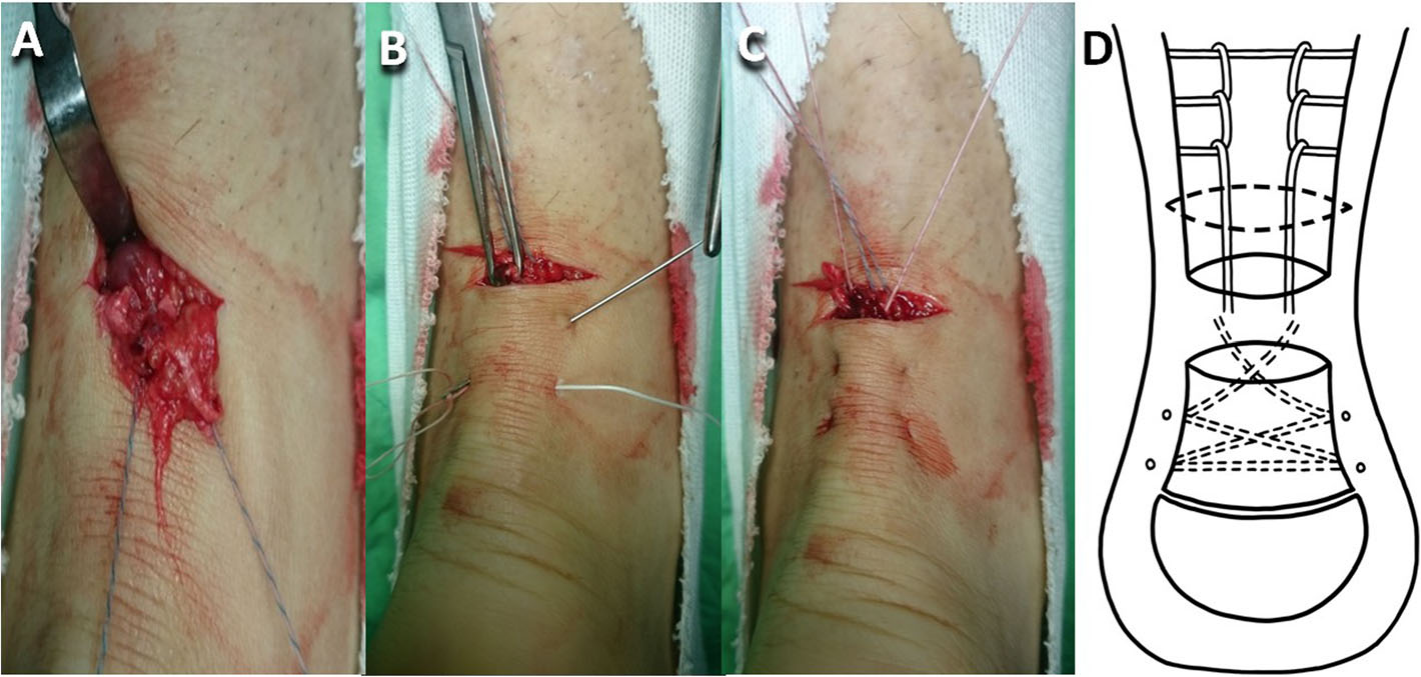
**a** Krackow locking loop sutures were applied at the proximal stump through a 3-cm transverse incision 2 cm proximal to the palpable tendon rupture gap. **b** The percutaneous suture was crisscrossed through the distal stump. **c** The end of the distal stump suture was subcutaneously passed through the transverse incision. **d** Illustration of (**a**)–(**c**)

**Fig. 2:**
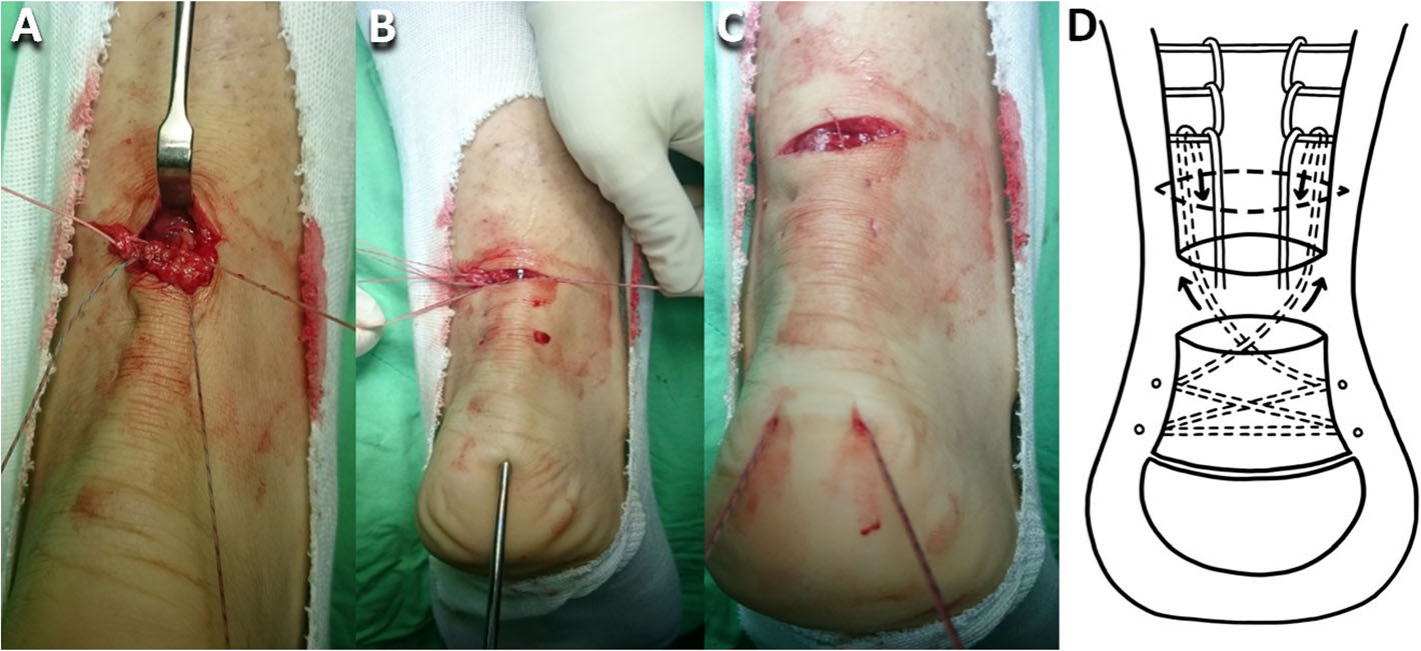
**a** The end of the distal stump suture was looped through the proximal stump Krackow locking loop as the pulley. **b** The bird-beak arthroscopic suture passer was passed subcutaneously through the heel mini-incision over the calcaneous tuberosity and out from the transverse incision. **c** The ipsilateral Krackow sutures and the contralateral crisscrossed sutures were subcutaneously pulled through the heel incision. **d** Illustration of (**a**)–(**c**)

**Fig. 3:**
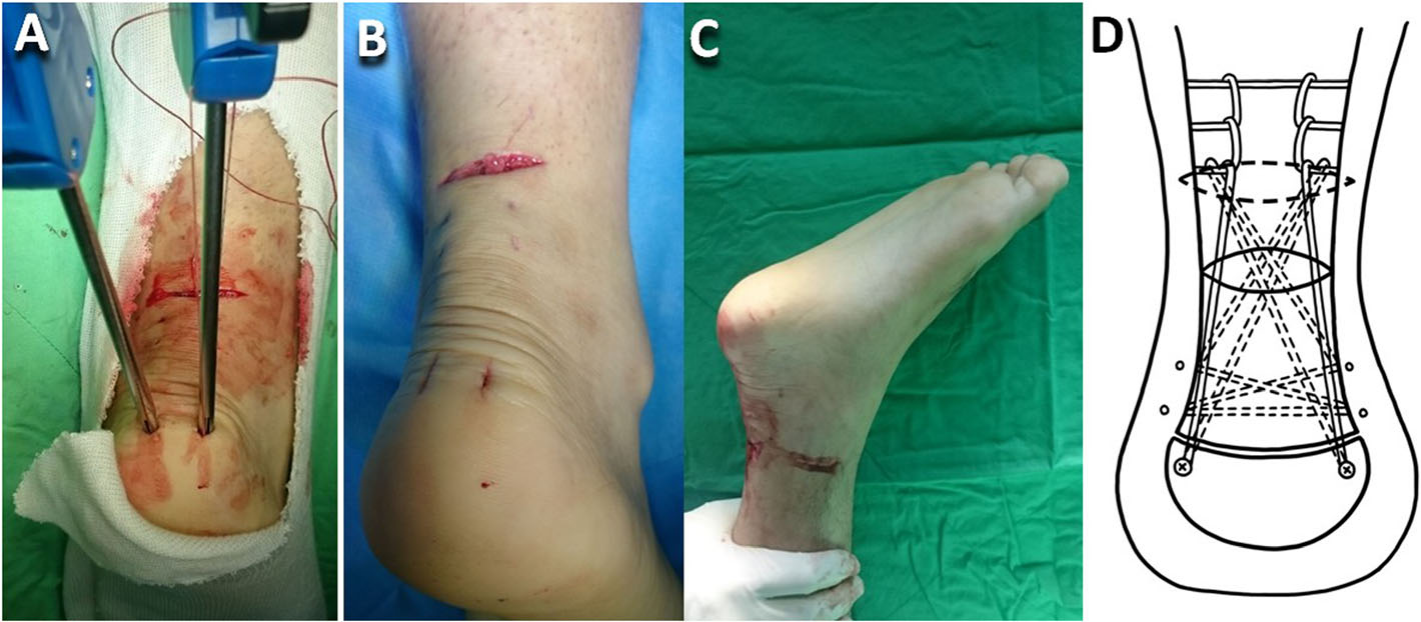
**a** The sutures were seated at the posterior of the calcaneal tuberosity with two 4.5-mm knotless suture anchors. **b** The epitenon was repaired. **c** The Achilles tendon tension was checked post-operatively with the leg erect. **d** Illustration of (**a**)–(**c**)

After a patient had been spinally anesthetized, they were put on the table in the prone position, and an air tourniquet was placed on the thigh. The tendon gap was palpated to identify the ruptured end. A 3-cm long transverse incision was made 2 cm proximal to the ruptured end. The proximal stump was gently pulled out through the transverse incision with the knee in flexion position after the stump had been freed from the surrounding paratenon and plantaris tendon, if it was present, using a 1-inch ribbon malleable retractor. The hematoma was completely debrided. Krackow locking loops were used on both sides of the soleus muscle and healthy tendon of the proximal stump (Hi-Fi® Suture; CONMED Corporate Headquarters, Utica, NY, USA) (Fig. 1a, d). An Allis clamp (forceps) was subcutaneously inserted through the transverse incision in maximum ankle plantar flexion to maintain the tension of the distal stump, and then the percutaneous suture was crisscrossed through the distal stump (CONMED) (Fig. 1b, d). The end of the distal stump suture was subcutaneously passed through the transverse incision (Fig. 1c) and then looped through the proximal stump Krackow locking loop as the pulley (Fig. 2a, d). Two 0.5-cm long vertical incisions were bilaterally made on the posterior calcaneal tuberosity, and then the bird-beak arthroscopic suture passer was subcutaneously passed from the vertical incision to the transverse incision (Fig. 2b, d). The subcutaneous tunnel must be empty to avoid skin dimpling in the subsequent suture passage. The ipsilateral Krackow suture end and contralateral crisscross suture end were passed down to the distal mini-vertical incision (Fig. 2c). The sutures were seated at the posterior calcaneal tuberosity with two 4.5-mm suture anchors (PopLok® Knotless Suture Anchors; CONMED) (Fig. 3a, d). The tendon rupture gap became smooth and impalpable when the sutures were pulled to symmetrically proper tension in 30° of knee flexion and ankle plantar flexion. The anchors were then locked. A 3-0 absorbable running stitch (Monocryl; Ethicon, Johnson & Johnson Medical N.V., Belgium) was used at the epitenon (Fig. 3b). The wound was irrigated and closed layer by layer with 3-0 and 4-0 monocryl subcutaneous sutures and finally closed with reinforced antimicrobial skin closures (Steri-Strips; 3 M Health Care, St. Paul, MN, USA). The Achilles tendon tension was checked with the leg erect immediately post-surgery (Fig. 3c).

### Rehabilitation protocol

Patients were advised to do an active ankle-pumping exercise, to do a non-weight-bearing range of motion exercise for at least 1 h a day, and to walk without a cast or splint protection, all immediately post-surgery. They were also advised to walk full weight bearing (FWB) with crutches and wearing shoes with an added heel wedge (3 cm). One-week post-surgery, patients were allowed to begin walking without ambulatory aids (canes, crutches, walkers, etc.). Two weeks post-surgery, we recommended that they reduce the added heel wedge height by 1 cm per week. Muscle power training with a concurrent heel-raising exercise began 1 month post-surgery, and 6 weeks later, patients were permitted to return to exercise as tolerable.

## Results

### Surgery-related data

The mean time between injury and surgery was 4.3 days (range, 1–9 days). The mean surgery duration was 22 min (range, 18–36 min). The transverse incision averaged 6.3 cm (range, 5.5–7 cm) proximal to the calcaneal tuberosity.

### Complications

**Fig. 4:**
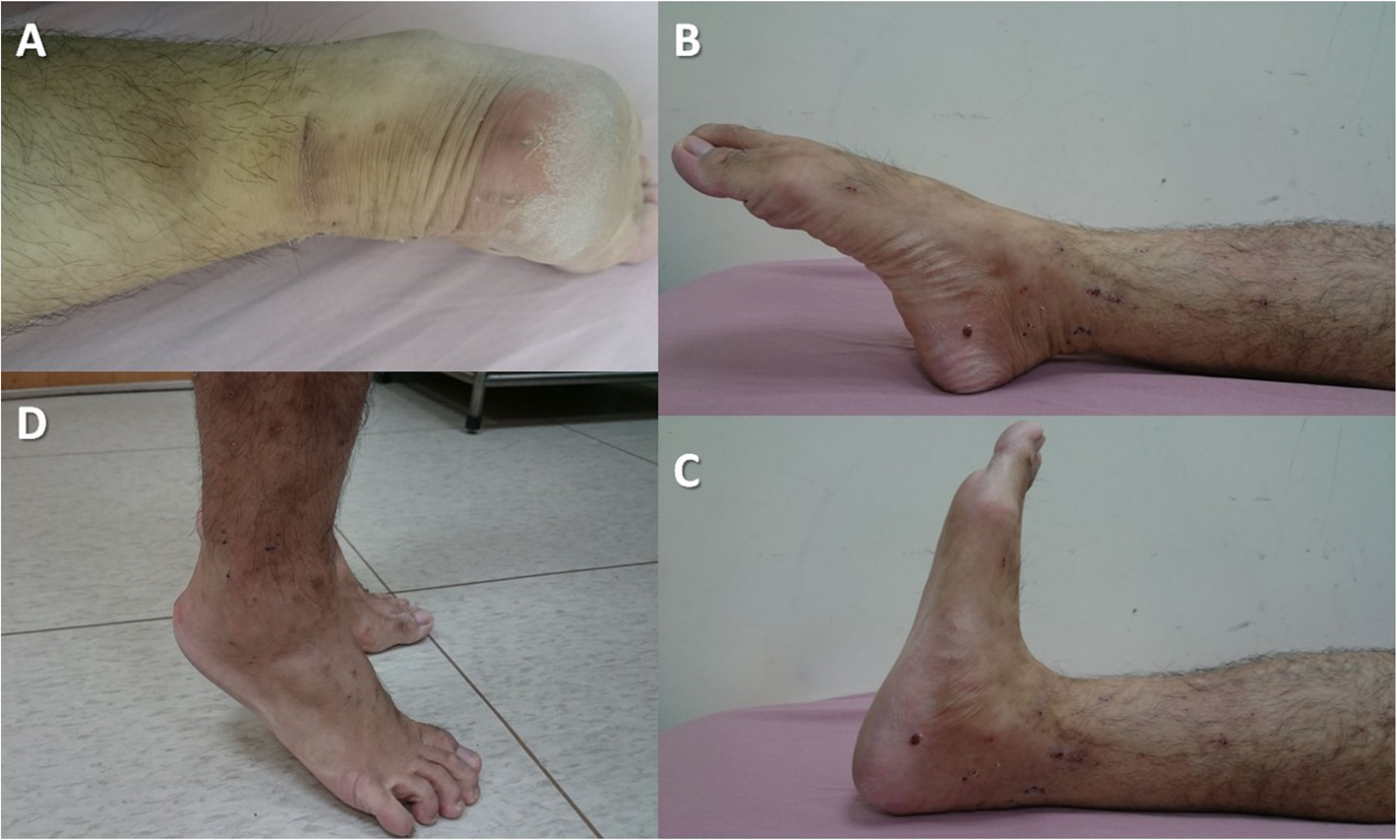
**a** The incision scar 4 weeks post-operatively. **b** Active plantar flexion and **c** active dorsiflexion 4 weeks post-operatively. **d** Heel-raise standing without aid 5 weeks post-operatively

There were no serious complications in the present study. All incisions healed well and without scar adhesions or superficial or deep infections (Fig. 4a). There were no sural nerve injuries, reruptures, deep vein thromboses, or pulmonary embolisms.

### Functional outcomes

After 1 post-operative year, all 10 patients scored 100 points on the American Orthopaedic Foot and Ankle Society (AOFAS) scale, and they returned to their preoperative exercise levels. Patients needed a mean of 7.5 days (range, 3–11 days) to walk FWB without crutches and they needed a mean of 24.5 days (range, 21–28 days) to walk with a full ankle-joint range of motion (Fig. 4b, c). To stand with a raised heel without an aid, they needed an average of 35 days (range, 28–42 days) (Fig. 4d). All patients returned to their jobs, which needed an average of 45.5 days (range, 35–63 days), and to their previous athletic exercise level, which needed a mean of 147.5 days (range, 84–210 days).

## Discussion

There is still no consensus about the best way to manage acute Achilles tendon ruptures [7–10]. In active young athletes who want to quickly return to competition, surgery is indicated to avoid muscle atrophy after non-surgical treatments [14]. Because of improvements in surgical techniques, multiple MIS methods have been developed, and laboratory studies have reported that tensile strength in the MIS group was comparable with that in the open surgery group [20]. A retrospective series [21] reported that MIS had returned patients to baseline physical activities significantly sooner than open repair did. However, sural nerve injury continues to be the major concern when the Achilles tendon is repaired using MIS [16–18].

A cadaver study [17] reported that the sural nerve crosses the lateral border of the Achilles tendon 8.28–8.96 cm proximal to the calcaneal tuberosity, which means that surgeons can usually avoid injuring the sural nerve if it is less than 8 cm proximal to the calcaneal tuberosity. Thus, our modified MIS used Krackow sutures at the proximal stump and percutaneous sutures at the distal stump.

In our series, the mean transverse incision was 6.3 cm (range, 5.5–7 cm) proximal to the calcaneal tuberosity, which corresponded with the common rupture sites and prevented iatrogenic injury of the sural nerve. Furthermore, one study [22] reported that the posterior longitudinal incision was in a less vascularized zone of the skin that covers the Achilles tendon. In our technique, the incisions were transversely proximal to the rupture site to prevent wounds located in less vascularized zones if the incisions are posterolongitudinal. Our patients had no incision complications. The transverse incisions were 2 cm proximal to the ruptured end; thus, we were able to gently pull out the proximal stump in the knee flexion position. The pulled-out stump with the healthy tendon part was long enough to let us use Krackow sutures, which are simple, commonly used, and strong enough to permit early post-operative rehabilitation [23].

Two other studies [10, 24] described the internal brace concepts in Achilles tendon repair and reported excellent outcomes. To preserve the proximal stump blood supply, these two studies made additional incisions at the gastrocnemius myotendinous junction. We, however, used Krackow sutures at the healthy tendon and additional soleus muscle. There were no reruptures in our case series; thus, the blood supply was not obstructed for tendon healing. Other internal brace techniques have been reported [25, 26], but those studies required specially designed suture jigs, which we did not.

Early ankle range-of-motion improved after early post-operative FWB walking [27]. Another study [28] reported a greater risk of ankle stiffness in the non-weight-bearing group. A recent meta-analysis [29] claimed that early functional rehabilitation improved patient satisfaction and facilitated an earlier return to normal everyday activity after Achilles tendon rupture repair than post-operative immobilization did. Furthermore, there were no significant differences in major complications between the two groups [29]. These findings, which agree with ours, indicate that early stretching and stressing of the repaired tendon improve functional outcomes. Variability in rehabilitation protocols, surgical repair techniques, and adopted functional outcome parameters yielded a variety of differing outcomes and complications (Table 1). However, more studies now emphasize early weight-bearing and ankle range-of-motion after adequate repair [10, 14, 25, 27]. Despite differences in the protocols in the above studies, there are no significant differences between our results.

**Table 1:** Literature review on post operation rehabilitation protocol and functional outcome in Achilles tendon rupture

Study	Year	Study type	Surgical method	Rehabilitation protocol	Functional outcome	Complication
Valkering KP et al. [27]	2016	Randomized control FWB **[**mobilized full weight bearing group**]** (*n* = 27) compared with IMM **[**immobilized non-weight-bearing group] (*n* = 29)	Longitudinal open incision; end to end repair with Kessler sutures	**FWB**: *0–2nd week*: FWB with adjustable orthosis and crutch; 15–30° range of motion in plantar flexion *3rd–6th weeks*: 5–45°range of motion in plantar flexion **IMM:** *0–2nd week*: non-weight-bearing with crutch; ankle immobilized in 30°of equinus position. *3rd–6th weeks*: FWB with crutch and wearing the heel added orthosis.	Improved early ankle range of motion (6 months); no difference in following 1 year	One patient in IMM group had traumatic rerupture.
Olsson N et al. [14]	2013	Randomized control	Surgical group (longitudinal wound incision, end to end repair with a modified Kessler technique) (*n* = 43) compared with non-surgical group (*n* = 45)	Surgical group: *0–2nd week:* ankle immobilized in a pneumatic walker brace with heel pads producing a plantarflexion approximately 20°. FWB with crutch was allowed. *3rd week*~: Early active range of motion and strength training. Non-surgical group: *0–2nd week*: The same as the surgical group. *3rd–8th weeks:* Immobilized in the brace for 8 weeks.	Surgical group was significantly superior in the drop counter movement jump and hopping in following 1 year. No significant differences between the groups in symptoms, physical activity level, or quality of life.	Six superficial infections in the surgical group.
Sarman H et al. [24]	2016	Retrospective analysis.	Semi-invasive internal splinting (SIIS group, *n* = 24) compared with open end to end repair with Krackow sutures (open group, *n* = 21)	Ankle immobilized in 30° plantar flexion with dorsal splint after operation. No further rehabilitation protocol was available in this article.	No significant differences between the groups in functional outcome in 1 year following.	One sural nerve injury in SIIS group (recovered 6 months later). Two deep wound infection in open surgery; one underwent debridement, and another one required additional soft tissue coverage.
Bevoni R et al. [8]	2014	Case series	Longitudinal open incision; triple-bundle technique (*n* = 66)	*0–2nd week*: non-weight-bearing with walking boot. *3rd week*: partial weight bearing with boot locked in neutral position *4th–5th weeks*: partial weight bearing with boot unlocked in in 20–30°of plantar flexion. *6th week*: partial weight bearing without boot *8th week*: full weight bearing	The mean American Orthopaedic Foot and Ankle Society scale score (AOFAS) at 36 months was 93.9 ± 5.9	One patient had a significant amount of scar adhesion.
McWilliam JR et al. [25]	2016	Case series	Internal brace (IB) with percutaneous Achilles repair system (PARS; Arthrex Inc., Naples, FL) (*n* = 34)	*0–1st week:* Crutch-aided FWB with walking boot with heel wedge; 1/4 wedge removed every two weeks. *2nd–3rd weeks:* FWB with boot only, active dorsiflexion of the ankle is allowed without passive dorsiflexion. *4th–5th weeks:* Passive dorsiflexion is allowed to neutral. *6th–7th weeks:* Remove boot *8th week:* Passive dorsiflexion beyond neutral.	The Achilles tendon total rupture score was 94 ± 14 in following range: 24–36 months	Nil
Yin L et al. [10]	2017	Case series	Panda rope bridge technique (*n* = 11)	*0–1st week:* Active range of motion without weight-bearing. *2nd–6th weeks*: FWB walking without crutches while wearing a 30-mm-height heel, which decreased 5 mm once a week. *7th–8th weeks*: muscle strengthening exercises. *9th week*: advised to take part in athletic exercises gradually	The mean AOFAS score at 12 months was 100.	Nil
Current study	2019	Case series	Jigless knotless internal brace technique (*n* = 10)	*0–1st week*: FWB with crutches and wearing shoes with an added heel wedge (3 cm); non-weight-bearing range of motion exercise at least 1 h a day. *2nd week*: walking without ambulatory aids was allowed. 3rd–5th *weeks*: reducing the added heel wedge height by 1 cm per week. *5th–6th weeks*: heel-raising exercise *7th week*: exercise as tolerable	The mean AOFAS score at 12 months was 100.	Nil

The present study appears to be the first to report using a jigless knotless internal brace to repair acute Achilles tendon ruptures. We found, after 1 year of follow-ups, that this simple technique was efficacious.

## Limitations

Our study has limitations. First, our sample was small: only 10 cases. Second, we did not compare our sample with a control group that had been treated using another repair technique. Third, the follow-up duration was short. Fourth, the number of complications might not be realistically representative because the analyzed sample was small. Further studies with larger samples, longer follow-ups, and a control group are needed to confirm our findings.

## Conclusion

Our jigless knotless internal brace technique is simple and was efficacious. Specially designed tools were unnecessary. There were few soft tissue complications. Functional recovery was facilitated because the blood supply to the Achilles tendon was preserved, and because of the strong suture structure. Thus, this technique might be a reliable option for repairing ruptured Achilles tendons.

## Data Availability

The datasets used and analyzed during the current study are available from the corresponding author on reasonable request.

## Abbreviations

AOFAS scale: American Orthopaedic Foot and Ankle Society scale
FWB: Full weight bearing
MIS: Minimally invasive surgery
